# Discrepancies between Self- and Clinical Staff Members' Perception of Cognitive Functioning among Patients with Schizophrenia Undergoing Long-Term Hospitalization

**DOI:** 10.1155/2019/6547096

**Published:** 2019-11-03

**Authors:** Fumiko Kaneko, Hitoshi Okamura

**Affiliations:** Graduate School of Biomedical and Health Sciences, Hiroshima University, Hiroshima 734-8551, Japan

## Abstract

In Japan, long-term hospitalization of patients with schizophrenia is still prevalent, even though the focus of psychiatric care is shifting from hospitals to the community. Difficulties in discharge planning often arise because clinical staff members' functional assessment differs from that of patients' self-assessment. Therefore, we attempted to identify characteristics related to these perceptual differences to promote the development of more effective approaches toward the discharge and societal reintegration of patients with schizophrenia undergoing prolonged hospitalization. Forty-eight long-term inpatients (23 men and 25 women with a mean age of 60.72 years) with schizophrenia were examined using the Schizophrenia Cognition Rating Scale Japanese version (SCoRS-J), Life Skills Profile (LSP), and Profile of Mood States- (POMS-) Brief Form. Differences between patients' self-ratings and clinical staff members' ratings on the SCoRS-J were used to divide patients into overestimators, underestimators, and accurate raters. These groups were then comparatively analyzed. Accordingly, overestimators displayed significantly severe cognitive dysfunction on the SCoRS-J objective ratings (*p* = .001) and significantly less difficulty on the SCoRS-J subjective ratings (*p* = .002) as compared to underestimators. Overestimators also scored significantly lower on the communication (*p* = .012) and responsibility (*p* = .021) LSP subscales compared to underestimators, and the total LSP score for overestimators was significantly lower compared to accurate raters (*p* = .036) and underestimators (*p* = .009). However, underestimators displayed significantly higher confusion on the titular POMS subscale than did overestimators (*p* = .021). These findings indicate that, among the three groups, overestimators scored lowest for objectively rated functioning. In contrast, underestimators attained the highest functioning; however, they were also confused. Clinical staff should examine how patients' self-perceptions deviate from the perceptions of staff and implement an appropriate approach considering the patient characteristics revealed from the results of this study.

## 1. Introduction

Psychiatric care in Japan has long been centered around hospitalization [1]. Consequently, many patients with psychiatric disorders, a substantial proportion of whom have schizophrenia, have been hospitalized for up to decades [2]. According to a survey conducted by the National Center of Neurology and Psychiatry in 2017 [3], the number of inpatients in psychiatric hospitals is approximately 284,000, of whom more than 55,000 (nearly 20%) are hospitalized for more than 10 years. Furthermore, there are nearly 26,000 inpatients who have been hospitalized for more than 20 years [3].

In 2004, the Japanese government formulated a vision for reforming mental health care and welfare based on the concept of moving “from a hospital-centric to a community-centric” model of care and began promoting rehabilitation interventions and community transition and integration from an early stage [4]. This has gradually reduced patients' average length of stay (LOS) in hospitals [5]. However, while this reduction reflects progress in the early discharge of new inpatients, many current inpatients with LOS extending from several years to several decades remain hospitalized [6]. The finding from a patient survey by the Ministry of Health, Labour and Welfare [7] that discharge rates decrease when LOS exceeds one year demonstrates that, in addition to encouraging the early discharge of new inpatients, it is also essential to prioritize ways to advance the release of current long-term inpatients [8]. The present study focused on the latter.

A wide range of factors make it difficult to plan the discharge of long-term inpatients with schizophrenia, including patients' personal issues, family problems, hospital environment, and community environment such as insufficient community-level assistance [9]. Poor insight [10, 11] and lack of self-confidence or poor self-efficacy in returning to community life [9, 10] are particularly important psychosocial factors impeding patients' discharge from hospitals.

Overconfidence resulting from unawareness of illness and poor insight or, conversely, low confidence even though objectively rated to have adequate capability suggest a divergence between patients' self-ratings and objective ratings by others concerning functional capacity or performance. This circumstance is encountered frequently in clinical settings and has been documented in many studies [12–20]. This divergence can often arise from patients' poor insight or self-assessment abilities. Patients with schizophrenia present with deficits in a wide range of cognitive functioning such as neurocognition [21, 22], social cognition [22, 23], and metacognition [23], which deteriorate their functional outcomes [21, 22] and social quality of life [21, 23]. These cognitive deficits, in combination with psychiatric symptoms and societal factors such as stigmatization, could affect insight and self-assessment in a reciprocal manner [18].

Furthermore, it has been reported that misestimation of ability is the strongest predictive factor for real-world functioning [19]. Harvey and Pinkham [20] stated that self-assessment of performance can be clinically helpful whether performance is objectively good or bad. Those with inferior performance could be helped to attempt to match their aspirations to accomplishments and improve over time, and good performers could have their functioning bolstered by recognizing their competence. Thus, the divergence between actual and self-perceived functional abilities is a key risk for patients' real-world functional outcomes. Furthermore, although it is vital for patients and clinical staff to share goals for discharge planning, a divergence in perception between these two parties can prevent agreement and make it difficult for clinical staff to provide discharge support. In addition, poor insight is related to lower compliance with medication and treatment [24–26], as well as lower interpersonal and social functioning [27–29]; therefore, improving insight is often a therapeutic target for patients with schizophrenia. However, increased insight is also associated with greater depression [30–33]; therefore, clinical staff members often fall into a dilemma between the benefits and risks arising from improving patients' insight.

Incidentally, this divergence in perception can be divided into two patterns: one where self-ratings are lower and the other where self-ratings are higher than the ratings by others. Differences in the divergence patterns and the presence or absence of divergence may be influenced by patients' characteristics. Bowie and colleagues [15] investigated the properties of these patterns of divergence among older outpatients with schizophrenia. Comparing patients' self-ratings of everyday real-world functioning with objective ratings by case managers, they subsequently categorized participants as accurate raters, underestimators, or overestimators based on their perceptual discrepancy scores; then, they performed a comparative analysis of the three groups. Accordingly, they found that accurate raters demonstrated greater social skills than did both overestimators and underestimators, while overestimators were the most cognitively and functionally impaired among the three groups. They also observed that underestimators had better cognitive skills and stronger tendencies toward depression than did overestimators.

Consistently, several other studies examined outpatients [17, 19]; however, this matter has not been thoroughly investigated regarding long-term inpatients with schizophrenia. Therefore, we examined the differences that characterize patterns of divergence in the perception of functional capacity between long-term inpatients with schizophrenia and clinical staff members. Our aim was to promote the development of better rehabilitation approaches toward discharge planning based on these patterns.

## 2. Materials and Methods

### 2.1. Participants

Patients who met the following criteria from six psychiatric hospitals in A Prefecture were included in the present study: (1) aged ≥ 20 years, (2) received a diagnosis of schizophrenia, (3) been hospitalized for ≥1 year, (4) presented with stable psychiatric symptoms (no restlessness) as confirmed by an attending physician, and (5) able to communicate verbally and consented to participate. Patients were excluded if they were judged by the attending physician as unable to understand the purpose of the study, questionnaire contents, or instructions owing to significant unawareness of their illness or reduced intellectual function.

In addition, for each patient, members of the attending clinical staff (nurses, occupational therapists, etc.) who best understood the patient's daily life were recruited as informants. Informant selection was left to the hospital staff.

### 2.2. Assessments

To assess cognitive functioning, everyday life skills, and mood states, several measures were administered to patients fulfilling the criteria stated above and to their attending clinical staff. All patients and informants were interviewed by the first author using the Japanese version of the Schizophrenia Cognition Rating Scale (SCoRS-J) [34] to evaluate patients' self-ratings of subjective difficulty and clinical staff members' ratings of objective severity in cognitive functioning. After receiving an explanation on how to evaluate the Japanese version of the Life Skills Profile (LSP) [35], informants were asked to complete it in order to provide an objective appraisal of everyday life skills. Further, patients were directly questioned using the Japanese version of the Profile of Mood States- (POMS-) Brief Form [36] to determine their subjective emotional status. Basic patient data such as age, sex, disease duration, and current LOS were also obtained from medical records and inquiries to clinical staff.

### 2.3. Instruments

#### 2.3.1. SCoRS-J

Developed by Keefe and colleagues [37], the SCoRS measures the severity of cognitive impairment in patients with schizophrenia with questions aimed at the degree to which this impairment affects day-to-day functioning through interviews of patients and their informants. It comprises 20 questions rated on a scale of 1 to 4, with higher scores indicating greater difficulty and severity. The Japanese version produced by Belvederi et al. [30] has been shown to be valid and reliable. Clinical staff members' ratings of objective severity using the SCoRS-J correlate with the total score on the Japanese version of the Brief Assessment of Cognition in Schizophrenia (BACS), a neuropsychological test for cognitive deficits.

In the present study, the difference between total objective and subjective values on the SCoRS-J (objective severity-subjective difficulty) was designated as the perceptual discrepancy score for disability in cognitive functioning. Perceptual discrepancy scores approaching 0 imply that patient and informant ratings are consistent, and patients can accurately self-assess their own cognitive functioning. Greater negative scores represent less self-estimation and suggest that patients feel more difficulties than the clinical staff members' ratings reveal, while greater positive scores represent more excessive self-estimation and suggest that the patient does not feel as impaired as implied by staff members' objective severity.

#### 2.3.2. Japanese Version of the LSP

Developed by Rosen and colleagues [38], the LSP measures the real-world, everyday living skills of patients with schizophrenia. Consisting of 39 items, the instrument evaluates five dimensions: self-care, nonturbulence, social contact, communication, and responsibility. Each item is rated on a four-point Likert scale, with higher scores demonstrating better life skills. The LSP is regarded as one of the optimal measures to evaluate the real-world functioning of patients with schizophrenia [39]. Clinical staffs were asked to assess their patients based on daily observations using a Japanese version of the LSP, which was produced by Hasegawa and colleagues [35].

#### 2.3.3. Japanese Version of the POMS-Brief Form

Developed by McNair and colleagues [40, 41], the POMS questionnaire evaluates six mood dimensions: tension-anxiety, depression-dejection, anger-hostility, vigor, fatigue, and confusion. Interviews were conducted using the Japanese version of the POMS-Brief Form, which consists of 30 items [36].

### 2.4. Analyses

After calculating basic statistical values from collected data, the Wilcoxon signed-rank test was applied to compare total SCoRS-J scores for objective severity and subjective difficulty and identify disparities in patients' and clinical staff members' ratings of disabilities in cognitive functioning. In addition, participants were divided into three groups based on perceptual discrepancy scores quantified by the differential between objective severity and subjective difficulty values on the SCoRS-J. Those with scores within ±0.5 standard deviations (SDs) of objective severity were designated as accurate raters, those with scores diverging from 0.5 SD of objective severity in the positive direction were designated as overestimators, and those with scores diverging greatly from 0.5 SD of objective severity in the negative direction were designated as underestimators. A one-way analysis of variance or the Kruskal-Wallis test was applied to detect differences among the three groups in each measured item. Items showing significant differences were then subject to multiple comparison analyses. Furthermore, a chi-squared test was used to examine differences in sex, marital status, and general work experience among the three groups. Statistical analyses were conducted with IBM SPSS Statistics for Windows, version 24 (IBM Corp., Armonk, N.Y., USA), and the two-tailed level of significance was set at 5%.

### 2.5. Ethical Considerations

The present study was conducted with approval from the Ethical Review Board of the Graduate School of Biomedical and Health Sciences, Hiroshima University.

The first author provided all participants with individual explanations of the purpose, method, and ethical considerations of this study. Written consent to participate in this study was obtained from the participants themselves.

## 3. Results

Forty-eight patients (23 men, 25 women; mean age = 60.72 years; age range = 31–77 years) participated.

Regarding SCoRS-J results, the Wilcoxon signed-rank test detected a significant disparity between clinical staff members' ratings of objective severity and patients' self-ratings of subjective difficulty (*p* = .046). Mean scores and SDs for perceptual discrepancy (i.e., the differential between objective severity and subjective difficulty values on the SCoRS-J) were −4.0 and 13.7, respectively. The least perceptual discrepancy was ±1. The largest positive perceptual discrepancy where objective severity surpassed subjective difficulty was +39, while the largest negative perceptual discrepancy where objective severity fell below subjective difficulty was −35.

The number of accurate raters with subjective difficulty within ±0.5 SD of objective difficulty was 14, while the number of overestimators and underestimators was 11 and 23, respectively.  Table 1 summarizes patients' sociodemographic characteristics, and Table 2 shows the mean scores and the results of the comparative analyses between groups for each rating scale.

**Figure 1:**
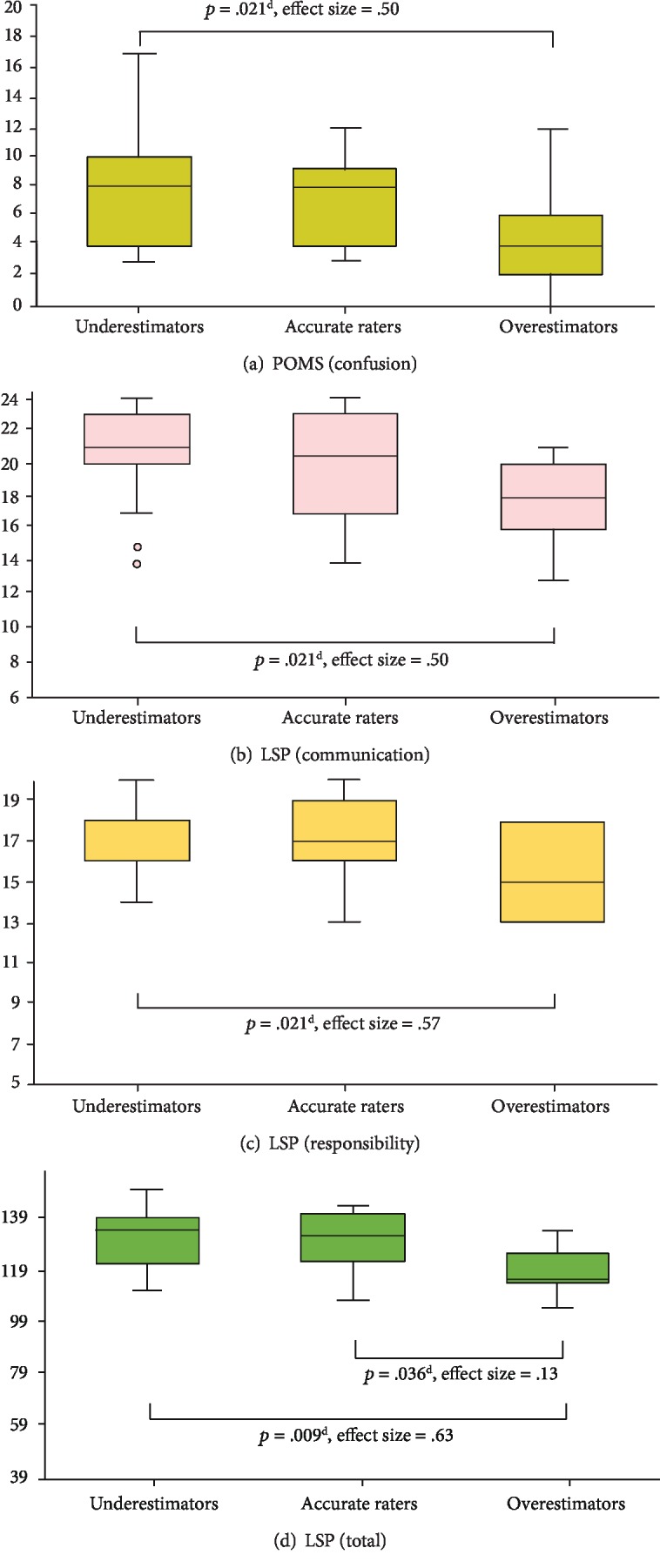
Items with significant differences among the three groups as determined by multiple comparisons. Note. ^d^Multiple comparisons with Bonferroni correction; POMS: Profile of Mood States; LSP: Life Skills Profile.

Regarding performance on instrument items, significant differences among the three groups were detected for the POMS subscale of confusion, the LSP subscales of communication and responsibility, and total LSP score. Multiple comparisons revealed that underestimators scored significantly lower than did overestimators on the individual items and that total LSP scores for overestimators were also significantly lower than those of either accurate raters or underestimators (Figure 1).

**Figure 2:**
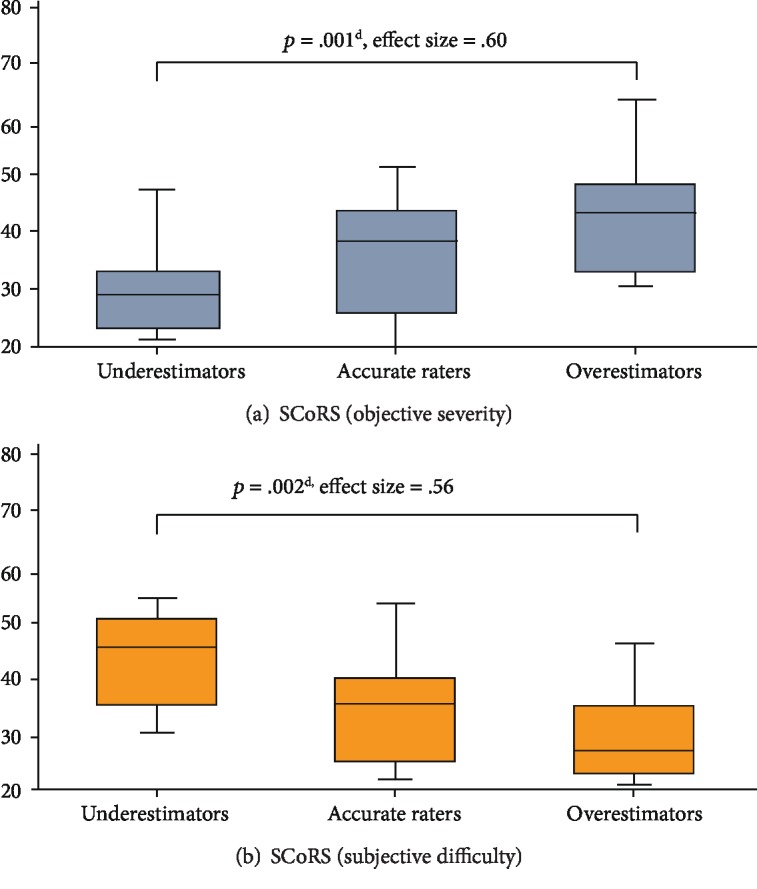
Comparison between objective and subjective ratings of cognitive functioning among the three groups. Note. ^d^Multiple comparisons with Bonferroni correction; SCoRS: the Schizophrenia Cognition Rating Scale.

When comparing the three groups regarding the differential between objective severity and subjective difficulty values on the SCoRS-J, objective severity ratings for underestimators were lower than those for either accurate raters or overestimators. In other words, although underestimators were objectively seen to experience disabilities in cognitive functioning to a lesser degree, their own ratings of subjective difficulty were much higher and significantly different from those of overestimators (Figure 2).

No significant differences were found among the three groups regarding sex, work experience, or marital history.

## 4. Discussion

The current results corroborate the findings of prior research that patients' self-ratings and clinical staff members' objective ratings of cognitive functioning diverge in many cases of schizophrenia. However, although previous studies encountered high rates of overestimators [15, 17, 19], we detected more underestimators. This may be because we examined long-term inpatients. Inpatients whose daily lives are managed under hospital rules and regulations are unable to live autonomously; therefore, living a prolonged passive existence may deprive patients of confidence in their abilities. Furthermore, hospitalization tends to lead to monotonous daily routines [42], and inpatients who function at a given level and have spent a long time under such living conditions may appear to cope with life without any problems. Accordingly, clinical staff may overlook potential difficulties these patients could be experiencing. Characteristic differences in patterns of divergence between self-ratings and objective ratings can be summarized as follows.

### 4.1. Accurate Raters with Minimal Perceptual Discrepancy

These patients tended to be older and more educated and had the highest onset age compared to the other two groups, albeit not to a level of statistical significance. They also showed the lowest frequency of hospitalization. However, their chlorpromazine equivalent dosage and scores on the tension-anxiety subscale of the POMS were greater than were those of the other two groups.

Compared to the other groups, they also achieved the best performance on the nonturbulence and social contact subscales of the LSP, suggesting that they were highly sociable. Such sociability may have been enhanced by their older age and longer years of education. This outcome supports the observations of previous studies by Bowie and colleagues [15] that accurate raters are associated with a high level of social skills and also indicates that proper awareness of one's abilities is a key factor for developing the capacity to observe collective rules, establish favorable relationships with others, and engage in social activities.

Further, their psychiatric symptoms were not inconsequential, as evidenced by their higher dosage of chlorpromazine equivalents; however, their symptom profile was believed to be unrelated to their competence in self-assessment, and they could maintain their social skills despite their condition. In contrast to their high social functioning, accurate raters experienced greater tension and anxiety, which insinuates that they feel stressed when they act socially or attempt to do so even though they experience psychiatric symptoms.

Although rates for hospitalization frequency and duration were lower compared to the other two groups, the present results alone cannot resolve the question of whether high social functioning mitigates hospitalization or vice versa. That said, it may be possible that patients who are adept at properly perceiving themselves are more likely to be discharged because they can cultivate their own personal motivations and goals.

### 4.2. Underestimators with Negative Perceptual Discrepancy Scores

This group included patients who reported difficulties in cognitive functioning due to cognitive disability to a greater degree than rated by clinical staff. Among the three groups, underestimators achieved the lowest objective ratings of disability in cognitive functioning and displayed high everyday living skills. They were evaluated the best on the self-care, communication, and responsibility LSP subscales, gaining significantly better results than did overestimators on the latter two subscales.

The communication subscale of the LSP measures verbal and nonverbal exchanges through factors such as starting or responding to conversation, refraining from intrusion into others' conversation, making eye contact, being difficult to understand because of disordered speech, talking about odd or strange ideas, and making expressions and gestures appropriate to the atmosphere. The responsibility subscale measures treatment compliance in general through factors such as being able to manage medication usage, proactively taking prescribed medications, and cooperating with health services by clinical staff. In other words, underestimators seem to have superior life skills particularly because they can take care of themselves and readily engage in daily conversations and because they are cooperative in treatment and diligently obey clinical staff members' instructions concerning proper use of medication and other matters. The present results suggest that although staff members may view such patients as capable individuals who merely lack confidence or motivation, it is possible that underestimators experience difficulties in everyday functioning due to cognitive disability to a greater degree than presumed by clinical staff.

Bowie and colleagues [15] reported that underestimators had significantly greater self-reported depression than did both overestimators and accurate estimators. The present study failed to detect a significant difference among the three groups regarding the POMS depression subscale but did observe a higher score for underestimators. However, a significant difference in the confusion subscale demonstrated that underestimators endure a greater level of psychological confusion compared to overestimators. Consequently, the possibility that clinical staff become biased because they are more inclined to develop positive emotions toward patients who are cooperative in treatment and encounter few problems in daily life cannot be ruled out. In other words, it can be inferred that staffs unwittingly begin to set unreasonable expectations in terms of skills and abilities and fail to recognize that their patients are distressed. Hence, it may be the case that situations where patients feel inept and unsure of themselves when others presume them to be capable could affect their mental states and intensify confusion. Alternately, it is also possible that patients become confused when matters “get out of hand,” even though they cannot pinpoint the exact cause of their problems. Judgments based on observational assessments and aspects of daily conversations will be insufficient to support such patients. Proper assistance will also require directly asking patients to describe the details of and their thoughts concerning troubles they encounter in daily life and conducting more suitable objective evaluations such as cognitive function and performance tests as necessary. Moreover, it is critical for clinical staff to develop a relationship with patients, through which they can explore solutions together.

### 4.3. Overestimators with Positive Perceptual Discrepancy Scores

Overestimators are unconcerned with their issues although they are objectively seen to have many problems. Such patients are regarded by clinical staff to be typical cases of poor reality testing. Among the three groups, overestimators exhibited the most severe disability in cognitive functioning and the lowest level of everyday living skills. Nevertheless, they were unaffected by mental confusion and led serene lives because they were unperturbed by the fact that they were objectively unable to accomplish many everyday tasks.

It is believed that self-awareness among overestimators was further reduced because, in addition to being affected by poor reality testing, they had become accustomed to long-term hospitalization and were living an existence where they did not have to face any daily inconveniences or difficulties. It is necessary to adopt an approach that provides such patients with opportunities to gradually acquire various experiences at a reasonable pace so that they can improve their reality testing through practical engagement. However, as illustrated by Sasaki and Yamada [11], long-term inpatients' level of satisfaction with their environment strongly affects their desire to be discharged. In many cases, those who lead sheltered and peaceful lives in hospitals and have become content with their status show no willingness to leave [11]. Hence, discharge planning approaches are extremely difficult, and clinical staff may be hesitant to provide support for discharge planning. Such patients cannot be released against their will, and returning elderly patients in marked physical decline to community life may be unrealistic. Nevertheless, clues to providing adequate assistance could be gained by carefully questioning patients about what happened during their long course of hospitalization that caused them to abandon their will to leave and what kinds of thoughts they had on the matter from time to time. In this manner, clinical staff should be counted on to steadfastly engage with such patients without giving up on listening to their voices.

Furthermore, factors that make it difficult to discharge long-term inpatients include not only personal factors, such as cognitive functioning and everyday living skills, but also social factors, such as family problems and inadequate community support. Therefore, it is essential to consider these social factors in addition to patients' personal issues when assisting long-term inpatients with discharge. Although the present study did not investigate in detail the social factors that resulted in long-term hospitalization of individual patients or those of patients without long-term hospitalization, in order to assist discharge more effectively for long-term hospitalized patients, it will be necessary to investigate their individual social factors and to take concrete measures to address them.

### 4.4. Limitations

The present study had several limitations. First, regarding determining cognitive functioning, we relied solely on the SCoRS-J, an interview-based coprimary measure, and no neuropsychological or performance-based tests were used. Such tests were excluded considering the burden they would place on the sizable proportion of elderly long-term inpatients who were examined. Since clinician assessments with the SCoRS-J have been confirmed to correlate with total scores on the Japanese version of the BACS, a primary rating scale of cognitive function, objective severity values generated by clinical staff are believed to reflect patients' degree of cognitive disability. However, for greater specificity, future research should consider employing objective tests and measurements that deliver a more detailed picture of cognitive levels and profiles.

Second, psychiatric symptoms and intellectual functioning could have influenced perceptual discrepancy. We included patients whose psychiatric symptoms were determined to be stable by a physician and excluded those with significant unawareness of illness and reduced intellectual capacity. However, it may be possible that diverse psychiatric symptoms, unawareness of illness, and intellectual capacity—even though they met the eligibility criteria—influenced perceptual discrepancies in some manner. Since we did not conduct assessments with instruments for psychiatric symptoms, administer intellectual tests, or collect data on unawareness of illness, the nature and degree of such influences cannot be identified. Moving forward, research should consider these issues when examining factors related to perceptual discrepancy in more detail.

Third, since the sample size of this study was small for comparing three groups, in future studies, it will be necessary to analyze a larger sample size.

## 5. Conclusion

The present study revealed that approximately 70% of participants had a divergence between patients' self-ratings and clinical staff members' objective ratings of cognitive functioning. Two-thirds of the participants who had a divergence were underestimators, and one-third were overestimators. We detected a higher rate of underestimators, which contrasted with previous studies on outpatients.

Furthermore, the psychological factor determining the characteristics of the group with different divergence patterns between self and objective perception was confusion rather than depression. This is another new finding different from previous study results. In order to provide more effective support for discharge, it is imperative that clinical staff and patients share rehabilitation goals. To that end, clinical staffs need to examine how their perceptions differ from patients' self-perceptions. Furthermore, better support should be provided by adopting approaches that recognize the key characteristic differences identified in the present study.

## Figures and Tables

**Table 1:** Comparison of sociodemographic characteristics between the three groups.

	All patients (*N* = 48)	Underestimators (*n* = 23)	Accurate raters (*n* = 14)	Overestimators (*n* = 11)	*p* value
	Mean (SD)	Mean (SD)	Mean (SD)	Mean (SD)	
Sex					
Male	23	12	6	5	.840^c^
Female	25	11	8	6	
Age (years)	60.72 (10.74)	59.91 (10.56)	64.21 (11.64)	58.00 (9.73)	.237^b^
Onset age of illness	25.66 (10.95)	22.59 (8.41)	30.27 (12.38)	25.50 (12.30)	.151^b^
Disease duration (years)	34.93 (12.88)	36.74 (13.11)	33.31 (12.23)	33.09 (13.83)	.653^a^
Current length of stay in hospital (months)	139.20 (133.28)	149.52 (139.98)	117.21 (141.80)	145.45 (115.43)	.284^b^
Number of hospitalizations	5.27 (4.79)	6.26 (5.15)	3.75 (3.31)	4.78 (5.38)	.350^b^
Education (years)	12.31 (2.42)	11.82 (2.22)	12.83 (2.86)	12.73 (2.37)	.246^b^
Marital history					
Presence	14	4	7	3	.105^c^
Absence	34	19	7	8	
Current marital status					
Presence	2	1	1	0	.673^c^
Absence	46	22	13	11	
Work experience					
Presence	26	11	8	7	.070^c^
Absence	14	10	4	0	
Unknown	8	2	2	4	
Chlorpromazine equivalents (mg)	757.86 (534.66)	664.20 (447.95)	883.64 (699.4)	793.64 (471.59)	.475^a^

Note: ^a^one-way analysis of variance; ^b^Kruskal-Wallis test; ^c^chi-square test.

**Table 2:** Mean scores and SD for each rating scale and comparison between the three groups.

	All patients (*N* = 48)	Underestimators (*n* = 23)	Accurate raters (*n* = 14)	Overestimators (*n* = 11)	*p* value
	Mean (SD)	Mean (SD)	Mean (SD)	Mean (SD)	
SCoRS					
Subjective difficulty	38.06 (11.09)	43.70 (10.21)	34.93 (9.93)	30.27 (8.21)	.002^b^
SCoRS					
Objective severity	34.52 (10.61)	29.17 (7.59)	36.29 (10.24)	43.45 (10.41)	.001^b^
POMS					
Tension-anxiety	6.16 (5.13)	6.35 (4.51)	7.93 (6.47)	3.55 (3.56)	.114^b^
Depression-dejection	4.35 (3.90)	4.65 (3.90)	4.50 (4.83)	3.55 (2.62)	.694^b^
Anger-hostility	5.29 (5.35)	6.17 (5.08)	4.21 (5.75)	4.82 (5.60)	.232^b^
Fatigue	5.60 (5.46)	6.91 (5.31)	5.43 (6.72)	3.09 (2.95)	.149^b^
Confusion	7.00 (3.59)	8.04 (3.74)	7.21 (2.89)	4.55 (3.14)	.025^a^
Vigor	7.43 (5.16)	6.61 (4.94)	8.86 (5.29)	7.36 (5.55)	.447^b^
LSP					
Self-care	30.35 (4.04)	31.30 (4.27)	30.79 (3.36)	27.82 (3.57)	.052^b^
Nonturbulence	43.52 (3.22)	43.48 (3.73)	44.43 (2.06)	42.45 (3.21)	.321^b^
Social contact	16.77 (3.42)	17.26 (2.77)	17.57 (3.99)	14.73 (3.38)	.073^b^
Communication	19.95 (3.12)	21.04 (2.85)	19.79 (3.29)	17.91 (2.55)	.015^b^
Responsibility	16.77 (2.03)	17.35 (1.82)	16.93 (1.98)	15.36 (2.01)	.024^a^
Total	127.37 (11.56)	130.43 (10.92)	129.50 (11.58)	118.27 (8.50)	.009^a^

Note: ^a^one-way analysis of variance; ^b^Kruskal-Wallis test. SCoRS: Schizophrenia Cognition Rating Scale; POMS: Profile of Mood States; LSP: Life Skills Profile.

## Data Availability

The data used to support the findings of this study are available from the corresponding author upon reasonable request.
